# Delusional Parasitosis in a School Teacher Living in a Rural Area: Parasitological Approach

**DOI:** 10.7759/cureus.22147

**Published:** 2022-02-12

**Authors:** Yassine Merad, Malika Belkacemi, Mounia Medjber, Derouicha Matmour, Zakaria Merad

**Affiliations:** 1 Cental Laboratory, Parasitology-Mycology, Hassani Abdelkader University Hospital, Sidi Bel Abbès, DZA; 2 Hemobiology and Blood Transfusion, Hassani Abdelkader University Hospital, Sidi Bel Abbès, DZA; 3 Psychiatry, Hassani Abdelkader University Hospital, Sidi Bel Abbès, DZA; 4 Central Laboratory, Therapeutic Chemistry, Hassani Abdelkader University Hospital, Sidi Bel Abbès, DZA; 5 Pathology and Laboratory Medicine, Hassani Abdelkader University Hospital, Sidi Bel Abbès, DZA

**Keywords:** skin disease/ dermatology, psychiatric disease, psychiatric symptoms, ekbom disease, delusional parasitosis

## Abstract

Delusional parasitosis is a psychotic illness. Patients often present to dermatologists with scars that are self-inflicted because they attempt to extract the “parasites”. We report a 58 -year-old female with an eight-month history of a crawling sensation on her skin and constant generalized itching, which she believed to be caused by insects and worms crawling across her skin. Examination revealed self-inflicted scratches at various stages of healing, which were limited to body parts within easy reach. The patient visited many physicians; it seems that she mutilated in an attempt to remove the offending organisms. She also presented skin scrapings and debris to her doctors, claiming that they contained worms and insects. Light pressure on the lesions did not produce any extrusion of macroparasites, and no parasites such as helminths and insect larvae (myiasis), were observed during microscopy. Thin smear scrapings were stained and examined to rule out parasitic diseases such as leishmaniasis and mycosis; however, no evidence of parasites was found. Our patient was administered with amisulpride 100 mg twice a day, which resulted in the complete remission of delusions after five weeks. The skin lesions were managed with clobetasol propionate ointment. A careful clinical examination combined with parasitological tests can be decisive in diagnosing delusional parasitosis, especially for patients from rural areas.

## Introduction

Ekbom's syndrome, formerly known as delusional parasitosis, is a relatively rare psychiatric disorder characterized by an inflexible and false belief of being infested with small organisms, insects, or parasites without any medical proof [1,2]. Patients firmly believe that they are infected with parasites despite lacking medical evidence [1] and become fixated on the perceived irritation [2]. This disorder is classified under non-schizophrenic delusions; however, it has also been reported to occur in schizophrenia, affective disorders, and organic or induced psychosis [3,4]. Delusional parasitosis is difficult to diagnose as many skin disorders cause itching, resulting in skin sores or irritation caused by scratching. The disorder most often presents as a monosymptomatic hypochondriacal psychosis in which no other thought disorders exist. Cooperation between dermatologists and psychiatrists, as well as antipsychotic treatments and psychotherapy, are the mainstays of the treatment. In our case, parasitological tests were performed to confirm the absence of parasitic infection and the presence of a psychiatric condition. 

## Case presentation

**Figure 1:**
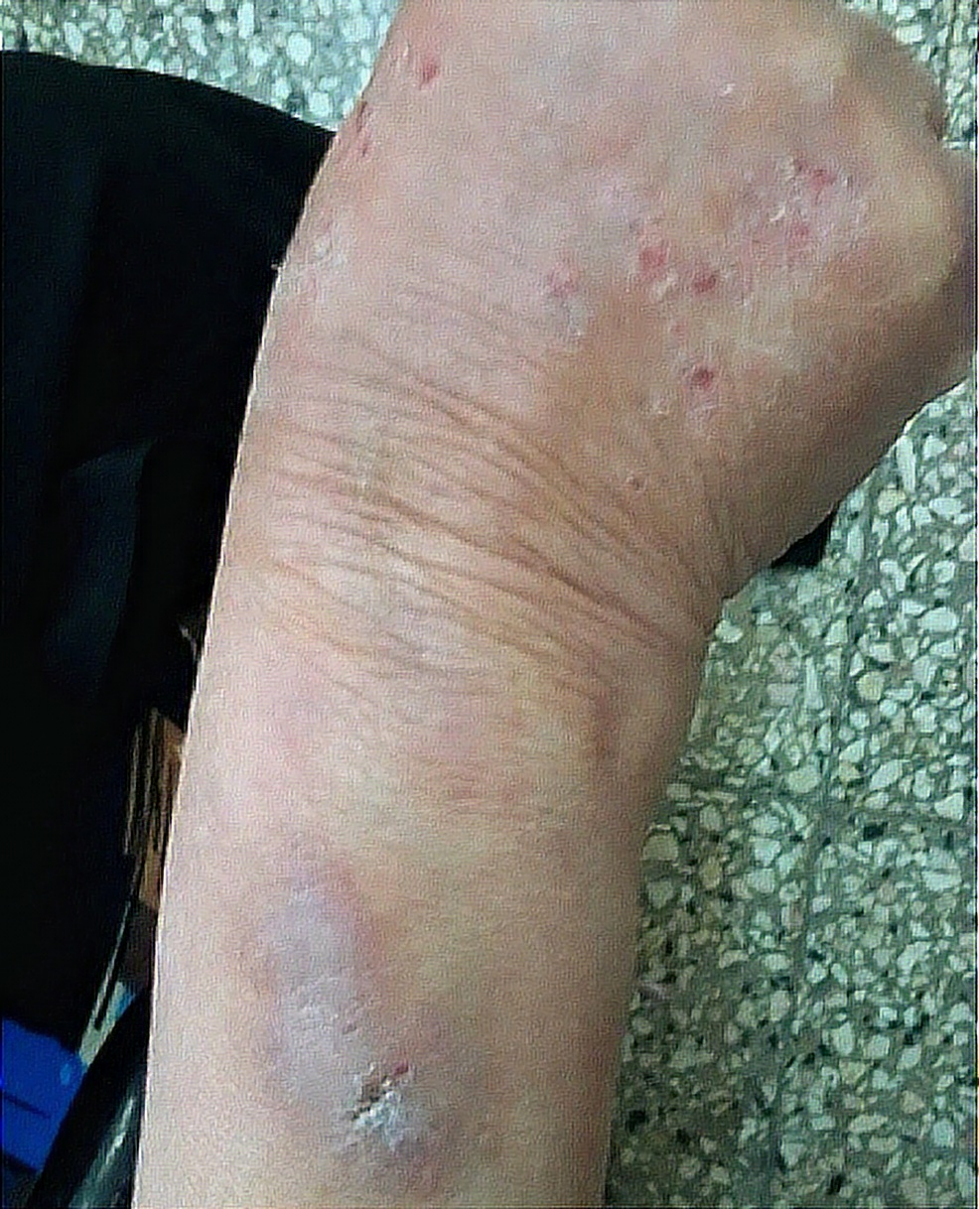
Skin lesions at various stages of healing and scars of different ages

**Figure 2:**
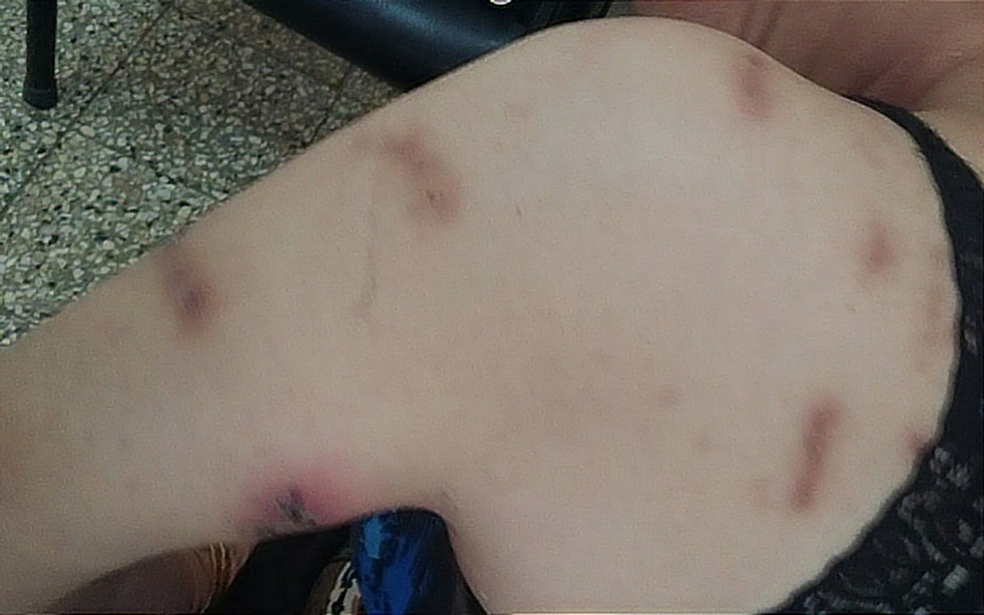
Arm and shoulder lesions at various sites (the scars indicate scratching on the side opposite the dominant hand)

**Figure 3:**
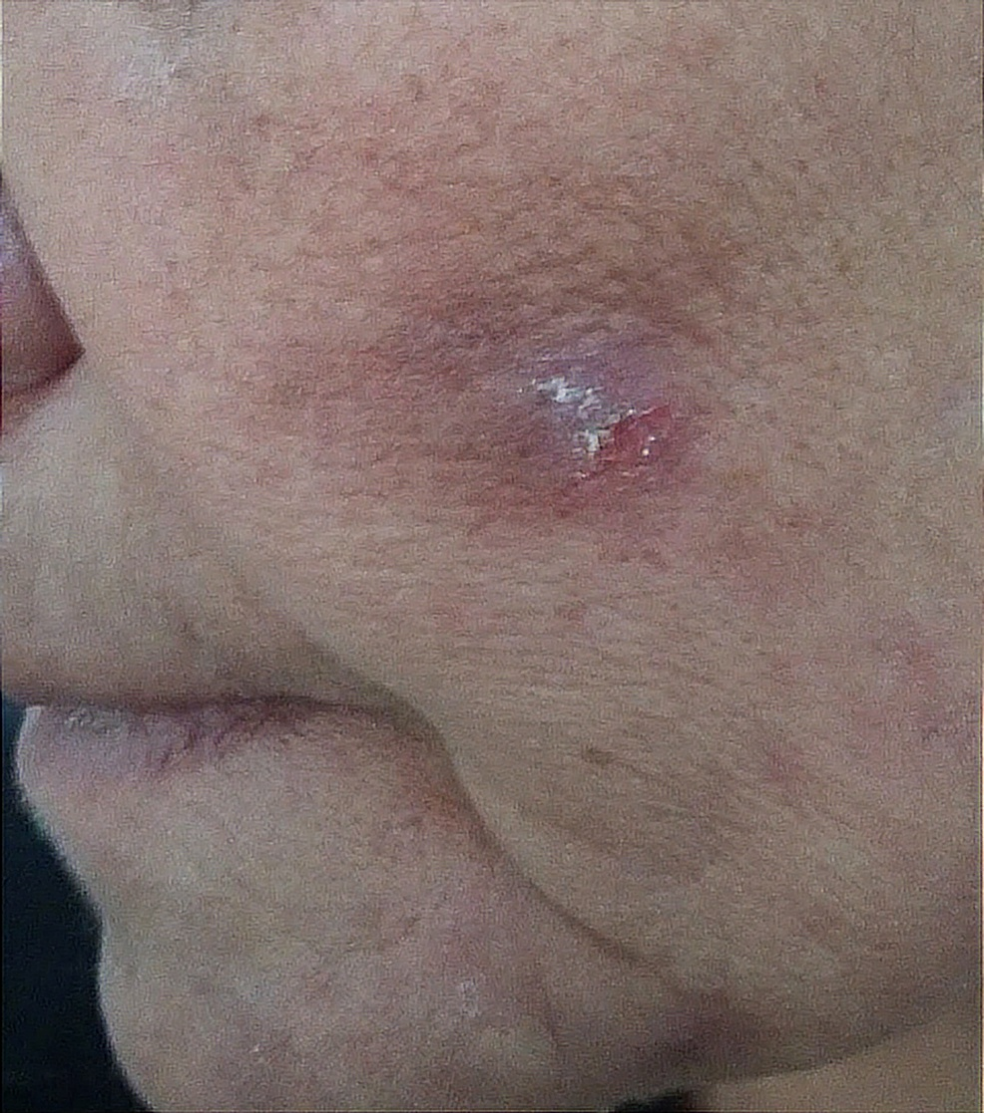
Self-inflicted lesion on the face

A 58-year-old female school teacher from a rural area was referred by the dermatology department owing to an eight-month history of a crawling sensation across her skin and itching throughout her body, especially on both arms and the left shoulder, which she assumed were due to insects and worms. Consequently, she had been showering numerous times a day with a copious amount of soap and had inflicted wounds on different body parts to extract the “parasites” (Figures 1-3).

During consultations with several physicians, she provided skin scrapings, thinking that they contained the parasites and worms. She firmly believed that these worms and insects were biting her and damaging her skin and that the itching and crawling sensations felt real to her. However, the detailed dermatological examination did not reveal any evidence of parasitic infestation. Hence, she was referred for further parasitological investigation by the Department of Parasitology-Mycology. Physical examination revealed several lesions on her arms, left shoulder, left cheek, and head. Light pressure on the lesions did not extrude any parasites or exudates. To rule out parasitic disorders, such as leishmaniasis and mycoses, serial scrape smears were taken from the lesions. Additional laboratory tests were also performed, including hemogram, urinalysis, stool exam, and thyroid function tests. While waiting for the microscopy and laboratory results, she was requested to attend a follow-up session after three days and to bring any worms or parasites extracted from her body. The microscopic examination did not reveal any parasites such as helminths, insects, or larvae. In addition, all the laboratory test results were normal. The results of the medical and biological investigations indicated that there was no convincing proof of a parasitic infection. Our patient was administered with amisulpride 100 mg twice a day, and skin lesions were managed with clobetasol propionate ointment twice a day. She was also given a list of local psychiatrists. These treatments led to complete remission after five weeks; no other skin lesions were observed thereafter. 

## Discussion

Ekbom’s syndrome, which is also known as acarophobia and parasitophobic neurodermatitis, was first termed “delusional parasitosis” by Wilson and Miller in 1946 [5]. Delusional parasitosis can be associated with different mental disorders, including schizophrenia, depression, anxiety, or obsessive-compulsive disorder. The patients have a firm but mistaken belief that they are infested with insects, fleas, worms, mites, lice, or other organisms [2]. Delusional parasitosis can be a shared psychotic disorder, as approximately 5%-15% of patients with delusional infestations share symptoms with other people, such as close relatives, partners, and family members [5,6]. Delusional parasitosis is a psychiatric disorder, classified as organic hallucinosis [7], affects women over 50, and occurs more frequently in people in lower socioeconomic levels [8,9]​​​. In such cases, antipsychotic medication is required [7]​​​​​​​. Continuous scratching is often experienced by patients, often resulting in bruises, skin ulcers, and scars as they attempt to extract the parasite [2,10]​​​​​​. Physicians may observe self-inflicted excoriations at various stages of healing that are limited to body parts within easy reach [2]​​​​. Usually, self-mutilation is considered pathological after the age of three [11]​​​​​​​. On examination, applying light pressure to the lesions may extrude eggs or feces; mechanical force and instruments can also be used to kill or catch the parasites [2]​​​​​​. However, careful removal of the infesting larvae remains the gold standard to confirm a parasitic infection and rule out a psychiatric disorder; a confirmatory skin biopsy is rarely needed. Patients often have several consultations with different physicians to seek relief and refuse to believe that their condition is a psychiatric disorder [3,12]​​​​​​​. To justify their belief, they often bring samples of hair, skin, and debris, such as dried scabs, dust, fibers, and lint, to the doctor to prove that the infestation is real; these often come in a matchbox, often called “Match-box sign” [1,13]​​​​​​​. The sizes of the samples are generally small, which probably contributes to the illusion of parasites and false interpretations [2]​​​​​​​. It is important to distinguish lesions due to arthropods, bacteria, and fungi from those of parasitosis. For patients in rural areas, parasitological tests may be necessary. A poor response to standard treatment for parasitic infestations should alert physicians of the possibility of delusional parasitosis. Our patient lives in a rural area, and cultural factors might have played a role in how she experienced and reported her symptoms. Antipsychotic drugs can be highly effective when treating delusional parasitosis; however, patients often refuse to accept their condition as a psychiatric disorder. Our patient was administered with amisulpride 100 mg twice a day which has resulted in complete remission after five weeks. An extensive literature review is necessary to better understand the clinical symptoms of this unique disorder.

## Conclusions

Delusional parasitosis is rare. The condition most often affects women over 50; patients often seek multiple consults because of a mistaken belief that they are infested with insects, worms, or other organisms. To justify this belief, they usually provide multiple skin scrapings and debris. Delusional parasitosis is difficult to diagnose as many skin disorders cause an itching sensation, which may result in skin ulcers or irritation. Nevertheless, patients with delusional parasitosis are fixated on the perceived irritation. Laboratory tests are required to rule out real parasitosis, especially in patients from rural settings. 
